# Interim analysis of all-case post-marketing surveillance study in Japan: lecanemab in patients with early Alzheimer’s disease

**DOI:** 10.1016/j.tjpad.2026.100541

**Published:** 2026-03-17

**Authors:** Atsushi Iwata, Yukinori Sakata, Kinuyo Koizumi, Akira Endo, Weijie Kuang, Kenta Sumitomo, Mika Ishii

**Affiliations:** a Tokyo Metropolitan Institute for Geriatrics and Gerontology, 35-2 Sakaecho, Itabashi-ku, Tokyo 173-0015, Japan; b Eisai Co., Ltd. 4-6-10 Koishikawa, Bunkyo-ku, Tokyo 112-8088, Japan

**Keywords:** Lecanemab, Amyloid-related imaging abnormalities, Infusion-related reactions, Post-marketing study

## Abstract

**Background:**

Lecanemab is a monoclonal antibody targeting amyloid-beta protofibrils, indicated for patients with mild cognitive impairment (MCI) and mild dementia due to Alzheimer’s disease.

**Objectives:**

This study reports interim findings of an ongoing, multicenter, prospective, observational post-marketing study for all patients treated with lecanemab in routine clinical practice in Japan, focusing on amyloid-related imaging abnormalities (ARIAs) and infusion-related reactions primarily observed during up to 28 weeks after treatment initiation.

**Methods:**

Patients treated with lecanemab at any medical institutions across Japan are included in the study. Data are collected using an electronic data capture system via standardized case report forms (CRFs). Study items included the incidence of ARIA, ARIA-edema or effusion (-E), ARIA-hemorrhage (-H: cerebral microhemorrhages, superficial siderosis, and macrohemorrhage), and infusion-related reactions, reported as adverse drug reactions.

**Results:**

As of July 5, 2025, CRFs from 2675 patients were collected, of whom 2672 had data available for the interim analysis. The median age was 76.0 years, and 62.6 % (1672/2672) of patients were diagnosed with MCI. At Week 28, 7.3 % (195/2672) of patients discontinued treatment, with a mean treatment duration of 189.6 ± 34.4 days. Among 2634 patients confirmed to have undergone MRI scans after treatment initiation, ARIA was observed in 7.1 % (188/2634) of patients, ARIA-E in 3.0 % (78/2634), and ARIA-H in 5.2 % (137/2634). Serious ARIA-H (macrohemorrhage) occurred in two patients (0.1 %). Infusion-related reactions were observed in 17.0 % (455/2672), including 0.7 % (18/2672) serious cases. The proportion of patients who experienced ARIA was highest in patients with apolipoprotein E (*APOE*) ε4 homozygotes.

**Conclusion:**

This interim analysis represents one of the largest real-world lecanemab cohorts reported globally to date. Although absolute rates are not directly comparable with those from clinical trials, the trends in ARIA distributions across *APOE* genotypes and infusion-related reactions were comparable to those observed in clinical trials.

## 1 Introduction

Alzheimer’s disease (AD) is a neurodegenerative disease that typically progresses through three main stages: preclinical AD, mild cognitive impairment (MCI), and dementia [1]. The preclinical stage is characterized by amyloid deposition (amyloidosis) in the cerebral cortex and cerebral blood vessels, followed by the formation of neurofibrillary tangles (tauopathy) [2,3]. MCI involves subtle cognitive decline but does not substantially interfere with daily activities [3]. Dementia represents the most advanced stage, with progressive impairments across multiple cognitive domains, particularly memory, and eventually interferes significantly with the ability to function at work or carry out daily activities [1]. In 2019, an estimated 51.62 million people were living with AD and other dementias worldwide, and among countries, Japan showed one of the fastest increases in incidence from 1990 to 2019 [4]. As the populations continue to age globally, therapeutic agents that slow or prevent AD progression are urgently needed.

Amyloid accumulation is one of the key pathological features of Alzheimer’s disease. Anti-amyloid-beta (anti-Aβ) monoclonal antibodies, particularly lecanemab and donanemab, have been shown to reduce brain amyloid burden and result in statistically significant cognitive benefits [[5], [6], [7]]. Currently, two amyloid-targeting therapies are commercially available, lecanemab [8] and donanemab [9]. Of these, lecanemab is a humanized IgG1 monoclonal antibody targeting Aβ that was approved in Japan in September 2023 for the treatment of MCI and mild dementia due to AD (thereafter, early AD) [8]. Among Aβ species that are considered pathological features of AD, lecanemab selectively binds to toxic [10], soluble Aβ protofibrils [11]. While effective, this class of therapies is also associated with an increased risk of amyloid-related imaging abnormalities (ARIAs) [[5], [6], [7]].

The safety and efficacy of lecanemab were evaluated in clinical trials. A phase 2b dose-finding global trial evaluated five lecanemab dose levels involving 854 participants with early AD. A well-tolerated 10 mg/kg biweekly dose was identified as the optimal dose [12]. An 18-month phase 3 global trial (Clarity AD, Core study) involving 1795 participants with early AD found that the participants treated with the optimal dose showed slowing of cognitive decline in the primary endpoint, as measured by the Clinical Dementia Rating-Sum of Boxes (CDR-SB), compared to placebo [6]. Key secondary measures of cognitive and functional assessments also demonstrated results in favor of lecanemab. The most common adverse events observed in the lecanemab group were infusion-related reactions (26.4 %), followed by ARIA-hemorrhage (-H) (17.3 %), ARIA-edema or effusion (-E) (12.6 %), headaches (11.1 %), and falls (10.4 %). Lower ARIA-E and ARIA-H incidence but generally comparable safety profiles were observed in the Asian subpopulation of Clarity AD [13].

As the clinical trials revealed commonly reported adverse events and the drug is likely to be administered to patients with diverse backgrounds in real-world clinical practice, an all-case post-marketing surveillance study was conducted in Japan to evaluate ARIAs and infusion-related reactions, treatment status, and long-term changes in cognitive and daily functioning. The present study reports findings from an interim analysis of this post-marketing study, with a focus on safety events of special interest, ARIAs and infusion-related reactions and treatment status, observed primarily during the 28 weeks after the initiation of lecanemab treatment.

## 2 Methods

### 2.1 Study design

This ongoing, multicenter, prospective, observational post-marketing study aims to evaluate the safety of lecanemab and observe long-term changes in cognitive function and activities of daily living (ADL) in all patients treated with this drug in routine clinical practice in Japan (ClinicalTrials.gov: NCT06322667). The conditions for lecanemab’s marketing approval were that, until sufficient data have accumulated, a post-marketing study targeting all patients is conducted to obtain background information on those receiving lecanemab, to collect safety and effectiveness data at an early stage, and to take necessary measures to ensure its appropriate use. The study is being conducted in compliance with the Ministerial Ordinance of Good Post-Marketing Surveillance Practice (GPSP Ordinance). The study protocol was reviewed and approved by Eisai Co., Ltd. for its scientific and ethical validity in accordance with the GPSP Ordinance. As this study is conducted under routine clinical practice, diagnosis of AD and disease severity (e.g., MCI, mild dementia due to AD), treatment plans, and adverse event assessments are made at the discretion of the attending physicians at participating institutions.

### 2.2 Patients and study settings

All patients treated with lecanemab at any medical institutions and departments in Japan will be included in this overall post-marketing surveillance study. There are no predefined exclusion criteria. The study was initiated on the date of launch in December 2023, and patient registration began concurrently and will continue through December 2026. This interim analysis targeted all patients treated with lecanemab from the launch to July 5, 2025 (data cutoff), and the analysis targeted all available data collected from CRFs 1 and 2 that had been locked.

### 2.3 Clinical setting

Generally, 10 mg/kg lecanemab (recombinant) is administered by intravenous infusion for approximately one hour every two weeks [8,14]. Precautions regarding the indications and efficacy include the requirement that the drug be used only in patients who have been diagnosed with AD and in whom findings suggestive of Aβ pathology have been confirmed using an approved diagnostic method, such as amyloid positron emission tomography (PET), cerebrospinal fluid (CSF) testing, or an equivalent diagnostic method. In addition, important precautions include monitoring of ARIA before the 5th, 7th, and 14th infusions, as well as periodically thereafter. Medical institutions that administer lecanemab are expected to ensure systems and resources including the following: trained personnel for MRI reading to detect ARIA, access to MRI scanners (≥1.5 Tesla), a structure in place to determine treatment continuation, discontinuation and to take appropriate measures when ARIA is detected, and staff capable of conducting cognitive assessments. In addition, each institution should have a system that allows prompt and appropriate management of adverse events.

### 2.4 Study procedures

Data are collected using an electronic data capture system via standardized case report forms (CRFs), and patients are registered centrally. After confirming each patient’s written or oral consent for the use of their data, the investigator proceeds with registration, preferably within 14 days of treatment initiation. Patients who receive the study drug before contracts with the participating institutions are finalized are subsequently registered, and data are collected retrospectively.

Each patient is followed-up for a maximum of 156 weeks (three years), which is defined as the observation period. Regardless of the treatment initiation date, the follow-up for all patients will be terminated by December 2027.

Treatment is defined as discontinued when lecanemab treatment is terminated before the end of the observation period, or if patients are lost to follow-up for reasons such as not visiting the medical institution. If an ARIA occurs and the patient’s outcome has not resolved at the time of discontinuation, the outcome is monitored until either recovery or 12 weeks after the onset of the adverse event, whichever occurs first.

CRFs will be collected in three separate volumes (CRF1, CRF2, and CRF3). CRF1 covers the observation period from the start of treatment to Week 28, whereas CRF2 and CRF3 cover the subsequent periods up to Weeks 80 and 156, respectively.

### 2.5 Study items and schedules

#### 2.5.1 Patient background

Baseline data include the following: sex, age, date of lecanemab treatment initiation, disease stage (diagnosis), prior treatment (lecanemab and other treatment for AD excluding the treatment for behavioral and psychological symptoms of dementia), MRI examination within one year before lecanemab initiation, apolipoprotein E (*APOE*) ε4 genotype testing (noncarriers, heterozygotes, homozygotes, etc.), Aβ pathology, and cognitive and functional assessment results (details described below). At the time the study was initiated, *APOE* testing was not covered by Japan’s national public health insurance system. Thus, *APOE* test findings are obtained from participating medical institutions if the test results have already been collected for clinical trials or other studies.

#### 2.5.2 Treatment status, concomitant medications, and disease stage

The data investigated from treatment initiation until the end of the observation period (or at discontinuation) include treatment status, concomitant medications (anticoagulants and antiplatelet/thrombolytic agents), and disease stage (diagnosis/severity [e.g., MCI, mild dementia due to AD]).

#### 2.5.3 Brain MRI examination

Brain MRI scans are assessed at baseline and through the end of the observation period (or at discontinuation), if performed according to the package insert [8]. The data include the date of examination, MRI parameters, and findings (ARIA-E, cerebral microhemorrhages, cortical superficial siderosis lesions, and intracerebral hemorrhage).

#### 2.5.4 ADRs

The safety events of special interest investigated until the end of the observation period (or at discontinuation) were ARIA-E, ARIA-H (cerebral microhemorrhages, superficial siderosis, and macrohemorrhage), and infusion-related reactions. Among these events, those for which a causal relationship with lecanemab cannot be ruled out are defined as adverse drug reactions (ADRs), and this interim analysis reports the ADRs. Information regarding symptoms, obtained during physician interviews with patients, was collected using open-ended fields, without a predefined checklist. Serious events were defined as death, life-threatening events, hospitalization or prolongation of hospitalization, persistent or significant disability or dysfunction, congenital anomaly or birth defect, or other medically important conditions. The events are monitored until the investigator determines that they are clinically resolved. In cases where an ARIA occurred and the outcome had not resolved, the outcome is monitored until either recovery or 12 weeks after the onset of adverse events, whichever occurs first. ADRs are classified according to the Medical Dictionary for Regulatory Activities Japanese Edition (MedDRA/J) version 28.0.

#### 2.5.5 Severity of dementia, cognitive functions, and ADL

The measures for assessing dementia severity, cognitive functions and ADL include the following: Clinical Dementia Rating [15,16], Mini Mental State Examination [17,18], Lawton’s Instrumental Activities of Daily Living [19], and Functional Activities Questionnaire [20]. Assessment data are collected at baseline and Weeks 28, 52, 80, 104, 132, and 156 after lecanemab treatment initiation (or at treatment discontinuation). The findings from these assessments will be provided in future reports.

### 2.6 Statistical analysis

The data included in this interim report were analyzed using descriptive statistics such as means ± standard deviation (SD) and median (minimum–maximum). ADRs were evaluated in the safety analysis set, which comprised patients whose CRF data were locked by the data cutoff and who provided consent for their data to be publicly disclosed, excluding those whose post-registration data were unavailable. Cumulative incidence of the first ARIA, ARIA-E, and ARIA-H was estimated using the Kaplan-Meier method for overall patients and for those whose *APOE* status was tested. All analyses were performed using SAS release 9.4 or later (SAS Institute, Inc., Cary, North Carolina, USA).

## 3 Results

### 3.1 Patient background

As of the data cutoff, 7936 patients from nationwide medical institutions were registered in 677 medical institutions for this study (eFigure 1). Of those, CRFs were collected from 2675 patients at the time of the data cutoff. Excluding three patients who were registered in error or lost to follow-up, 2672 patients for whom CRF1 was collected (including 16 patients for whom CRF2 was collected) were included in the safety analysis set for this interim analysis.

Of the 2672 patients, 65.6 % (1752/2672) were female (Table 1). The median age was 76.0 years (39–92), and the mean body weight was 53.5 ± 10.4 kg. Among these, 62.6 % (1672/2672) were diagnosed with MCI. Amyloid positivity was determined using PET in 64.2 % (1716/2672), CSF Aβ_1–42_/Aβ_1–40_ ratio in 34.4 % (918/2672), and both methods in 0.4 % (10/2672). *APOE* ɛ4 status was tested in 20.8 % (555/2672) of patients, and among those tested, 44.1 % (245/555) were heterozygous and 11.0 % (61/555) were homozygous.

**Table 1:** Patient background.

**Characteristic**	**Category**	**Safety analysis set**	
		**(*n* = 2672)**	
		**n**	**(%)**
Sex	Male	920	(34.4)
	Female	1752	(65.6)
Age (year)	<65	301	(11.3)
	≥65	2371	(88.7)
	Mean ± SD	74.3 ± 7.6	
	Median (min–max)	76.0 (39–92)	
Body weight (kg)	Mean ± SD	53.5 ± 10.4	
	Median (min–max)	52.0 (30.3–96.8)	
Clinical subgroup	MCI	1672	(62.6)
	Mild AD	1000	(37.4)
Previously treated for AD, excluding the treatment for BPSD	Treated	1254	(46.9)
	Not treated	1406	(52.6)
	Missing	12	(0.4)
Diagnostic methods for Aβ	Aβ PET imaging	1716	(64.2)
	CSF biomarkers	918	(34.4)
	Both Aβ-PET and CSF biomarkers	10	(0.4)
	Aβ negative	7	(0.3)
	Unknown or other	9	(0.3)
	No Aβ pathology assessment performed	12	(0.4)
*APOE* ε4 status	No tests performed	1972	(73.8)
	Tested	555	(20.8)
	Noncarrier*	235	(42.3)
	Heterozygotes*	245	(44.1)
	Homozygotes*	61	(11.0)
	Not shared the result*	12	(2.2)
	Waiting for the result*	2	(0.4)
	Missing	145	(5.4)

**Abbreviations:** SD, standard deviation; min, minimum; max, maximum; MCI, mild cognitive impairment; AD, Alzheimer’s disease; BPSD, Behavioral and Psychological Symptoms of Dementia; Aβ, amyloid- β; PET, positron emission tomography; CSF, cerebrospinal fluid; *APOE*, apolipoprotein E.

Notes:

Data are presented as frequencies and percentages unless otherwise indicated.

⁎ The denominator is the patients whose *APOE* ε4 status had been tested.

### 3.2 Treatment status

The overall mean treatment duration was 189.6 ± 34.4 (1–374) days, and the mean among patients who discontinued treatment was 105.4 ± 84.0 (1–374) days. At Week 28, 7.3 % (195/2672) of patients discontinued lecanemab treatment (Table 2). The reasons for discontinuation included patient preference (27.7 %, 54/195), adverse events other than safety events of special interests (26.7 %, 52/195), transfer to another hospital (18.5 %, 36/195), adverse events related to ARIA-E and ARIA-H (12.3 %, 24/195) and infusion-related reactions (11.3 %, 22/195), and disease progression (11.3 %, 22/195).

**Table 2:** Treatment status and duration.

	**Safety analysis set**	
	**(*n* = 2672)**	
	**n**	**(%)**
Treatment duration (day)	*n* = 2672	
Mean ± SD	189.6 ± 34.4	
Median (min–max)	197.0 (1–374)	
Treatment duration among patients who discontinued (day)	*n* = 211	
Mean ± SD	105.4 ± 84.0	
Median (min–max)	99.0 (1–374)	
Treatment status at Week 28		
Continued	2477	(92.7)
Discontinued	195	(7.3)
Reason for discontinuation at Week 28*		
Patient preference	54	(27.7)
Adverse event other than ARIA or infusion-related reactions	52	(26.7)
Transfer to another hospital	36	(18.5)
ARIA-E or ARIA-H	24	(12.3)
Other	23	(11.8)
Infusion-related reactions	22	(11.3)
Disease progression	22	(11.3)
Discontinued clinic visits during the course of treatment	13	(6.7)

**Abbreviations:** SD, standard deviation; min, minimum; max, maximum; ARIA-E, amyloid-related imaging abnormalities with edema or effusions; ARIA-H, ARIA with cerebral microhemorrhages, superficial siderosis, and macrohemorrhage.

Notes:

Data are presented as frequencies and percentages unless otherwise indicated.

⁎ Multiple reasons can be selected per patient.

### 3.3 Safety events of interest

**Fig. 1:**
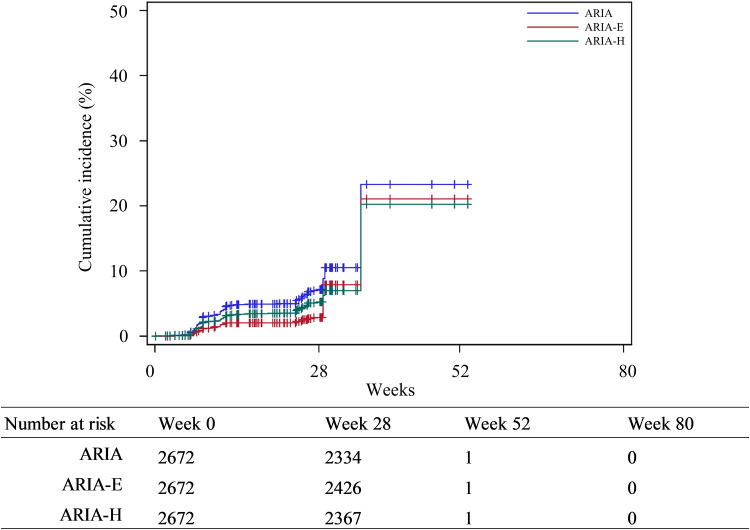
Cumulative incidence of first ADR event: ARIA, ARIA-E, and ARIA-H. **Abbreviations:** ADR, adverse drug reaction; ARIA-E, amyloid-related imaging abnormalities with edema or effusions; ARIA-H, ARIA with cerebral microhemorrhages, superficial siderosis, and macrohemorrhage.

Among 2672 patients, 2634 were confirmed to have undergone MRI scans after treatment initiation (Table 3). Of the remaining 38 patients without MRI scan data, the majority (89.5 %, 34/38) discontinued treatment within 60 days of initiation. ARIA occurred in 7.1 % of patients (188/2634). ARIA-E was observed in 3.0 % (78/2634), and the majority of the patients were asymptomatic. No cases were serious. ARIA-H was observed in 5.2 % of patients (137/2634). Cerebral microhemorrhage was observed in 4.9 % (129/2634). Intracerebral hemorrhage ≥1 cm (macrohemorrhage) was observed in two patients (serious cases), and their treatment was interrupted or discontinued. However, the majority of patients were asymptomatic and non-serious. ARIA-E and ARIA-H coexisted in 27 patients (1.0 %, 27/2634). Cumulative incidence curves for ARIA, ARIA-E, and ARIA-H are shown in Fig. 1.

**Table 3:** ARIA-E and ARIA-H.

**ADR**	**Safety analysis set**	
	**(*n* = 2672)**	
	**n**	**(%)**†
Patients confirmed to have undergone brain MRI examination after lecanemab treatment*	*n* = 2634	
ARIA-E or ARIA-H	188	(7.1)
ARIA-E	78	(3.0)
Asymptomatic	70	(2.7)
Symptomatic	8	(0.3)
Severe	0	(0.0)
Severity based on MRI findings		
Mild	47	(1.8)
Moderate	30	(1.1)
Severe	1	(0.04)
Serious	0	(0.0)
Non-serious	78	(3.0)
ARIA-H	137	(5.2)
Microhemorrhage	129	(4.9)
Superficial siderosis	10	(0.4)
Macrohemorrhage‡	2	(0.1)
Asymptomatic	133	(5.0)
Symptomatic	4	(0.2)
Severe	0	(0.0)
Severity based on MRI findings§		
Mild	123	(4.7)
Moderate	9	(0.3)
Severe	6	(0.2)
Serious	2	(0.1)
Non-serious	136	(5.2)
Both ARIA-E and ARIA-H¶	27	(1.0)
Coexisting of ARIA-E and ARIA-H#	27	(1.0)
Isolated ARIA-E	51	(1.9)
Isolated ARIA-H	110	(4.2)

**Abbreviations:** ARIA-E, amyloid-related imaging abnormalities with edema or effusions; ARIA-H, ARIA with cerebral microhemorrhages, superficial siderosis, and macrohemorrhage; ADR, adverse drug reaction; MRI, magnetic resonance imaging.

Notes:

⁎ Even if it was not possible to confirm brain MRI examination was performed for patients after the treatment initiation, patients were included if they had developed ARIA.

† The denominator is the patients described in the *.

‡ In Japan, the regulatory guidance has made it mandatory for cerebral macrohemorrhage to be included in ARIA-H.

§ Numbers indicate event counts, and multiple events per patient are included.

¶ Patients who experienced at least one incidence of both ARIA-E and ARIA-H (not necessarily simultaneously).

# Patients who experienced ARIA-E and ARIA-H simultaneously.

ARIA stratified by *APOE* ε4 status is shown in eFigure 2. In general, the proportion of patients who experienced ARIA, ARIA-E, and ARIA-H was the highest in *APOE* ɛ4 homozygous patients, followed by heterozygous patients and noncarriers (eFigure 2). Cumulative incidence curves for ARIA, ARIA-E, and ARIA-H stratified by *APOE* ε4 status are provided in eFigure 3.

**Fig. 2:**
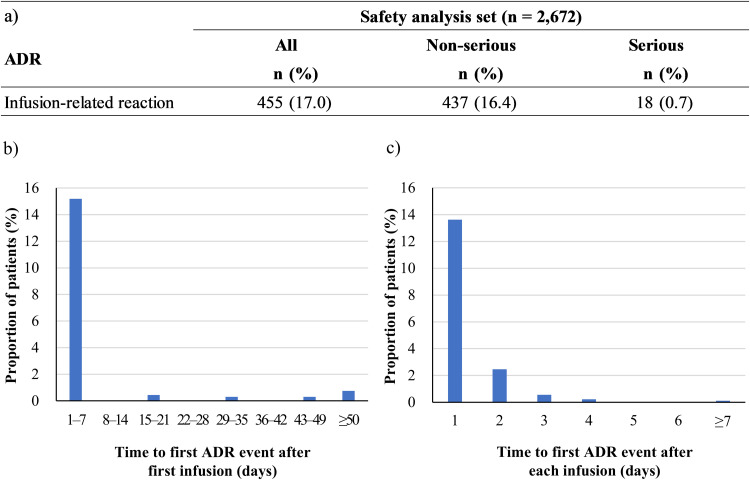
Infusion-related reactions: a) patients experienced infusion-related reactions, time to first ADR event after b) first infusion and c) each infusion. **Abbreviations:** ADR, adverse drug reaction.

Infusion-related reactions occurred in 17.0 % of patients (455/2672), of which 18 were serious (Fig. 2a). All 18 cases either recovered or improved. The most common symptoms were as follows: fever (11.3 %, 303/2672), headaches (3.4 %, 90/2672), chills (2.8 %, 74/2672), and fatigue (1.8 %, 47/2672). Anaphylactic reactions were not observed. The majority of the first infusion-related reactions occurred within seven days following the first infusion (15.2 %, 406/2672), and the events sharply declined with subsequent infusions (Fig. 2b). Similarly, across all infusions, events typically occurred on the day of infusion (13.6 %, 364/2672) (Fig. 2c). Among 455 patients who experienced an IRR, a total of 683 events (first and recurrent) were observed, and approximately 80 % of the events resolved or improved within three days: 25.8 % in one day, 40.8 % in two days, 13.6 % in three days, 5.7 % in four days, and 3.2 % in five days.

## 4 Discussion

This interim analysis of post-marketing study reported ARIAs, infusion-related reactions, and treatment status in 2672 patients from medical facilities nationwide, representing the largest real-world cohorts reported worldwide to date. Over an average of 189.6-day lecanemab treatment, ARIA occurred in 7.1 % of patients, and ARIA-E and ARIA-H occurred in 3.0 % and 5.2 %, respectively. Infusion-related reactions were observed in 17.0 %. Serious ARIA-H was observed in two patients (0.1 %), and serious infusion-related reactions occurred in 18 patients (0.7 %), but the majority of the cases were asymptomatic and non-serious.

The interim safety analysis set comprised 65.6 % females, with the mean age of 74.3 ± 7.6 years and MCI in 62.6 % of the patients. Patient characteristics were generally comparable to those of Clarity AD [6] and real-world studies conducted in the United States [21,22] and Israel [23]. Of note, *APOE* ɛ4 status was not confirmed in approximately 80 % of patients because, as mentioned above, *APOE* testing was not covered by national insurance at the time the study was initiated. However, among those whose status was confirmed, 55.1 % were *APOE* ɛ4 carriers, 44.1 % were *APOE* ɛ4 heterozygous and 11.0 % were homozygous. The overall carrier rate in the interim analysis cohort was slightly lower than that in previous reports, including Clarity AD (68.9 %) [6] and real-world studies (approximately 61 % [21] and approximately 64 % [22]). These discrepancies may be attributable to the limited proportion of patients who underwent *APOE* testing in our study (20.8 %) compared with previous studies.

The majority of patients were on lecanemab treatment at Week 28, and 7.3 % discontinued treatment. As we report interim findings, a direct comparison with previous reports cannot be made, and these findings need to be carefully interpreted. However, the overall discontinuation rate at this point of interim analysis was lower than that reported in the Clarity AD trial (18.8 % over 18 months) [6], but a closer rate was observed in a real-world study conducted in the United States (9.8 % over an average of 6.5 months) [21]. The reasons for discontinuation included patient preference, adverse events other than ARIA or infusion-related reactions, adverse events related to ARIA and infusion-related reactions, and disease progression. These reasons generally align with those observed in previous reports [6,21,23], but data regarding discontinuation due to disease progression are currently unavailable.

Over a mean 189.6-day lecanemab treatment period, the proportion of patients who experienced ADR ARIA was 7.1 %, and that of ARIA-E was 3.0 %. ARIA-E cases were typically asymptomatic and all were non-serious. As the study design including follow-up duration and reporting items (e.g., adverse events vs. ADRs) differ from those of previous studies, direct comparison cannot be made, and we report here the findings from previous studies. In an 18-month phase III Clarity AD [6], ARIA-E was observed in 12.6 % (113/898) of participants. The Asian subpopulation of Clarity AD reported lower numbers, with ARIA-E in 6.2 % (9/146) [13]. There are also reports from real-world studies, and among patients who received ≥4 lecanemab infusions in the United States, ARIA-E was observed in 15 % (29/194) over an average 6.5-month treatment [21]. Additionally, an interim report from a two-year retrospective study in the United States found that ARIA-E was observed in 7.9 % (14/178) of patients during a mean treatment duration of 375.4 days [24]. Similarly, a multicenter real-world study in China reported an ARIA-E incidence of 5.0 % (16/321) over a mean 5.6-month follow-up [25]. In both clinical trials and real-world studies, ARIA-E (with or without ARIA-H) mostly occurred within the first two to six months of treatment [6,21].

ARIA-H as an ADR occurred in 5.2 % of patients during the mean 189.6-day treatment period, including two cases of macrohemorrhage (0.1 %). However, the majority of ARIA-H cases were asymptomatic and non-serious. In Clarity AD, the incidence of ARIA-H was 17.3 % (155/898), with slightly lower numbers again in the Asian subpopulation (14.4 %, 21/146) [13]. Isolated ARIA-H, in contrast to ARIA-E, tended to occur throughout 18 months [6], and further data will enable the assessment of whether the trends observed in our real-world study are consistent with those reported in clinical trials and real-world studies.

We reported ARIA in relation to *APOE* status among patients whose *APOE* genotype was confirmed (approximately 20 %), and the proportion of patients who experienced ARIA, ARIA-E, and ARIA-H during follow-up was all numerically higher in *APOE* ɛ4 homozygotes followed by heterozygotes and noncarriers. Although absolute rates cannot be directly compared to those from clinical trials, the trends in ARIA distributions were generally in line with those from Clarity AD [6] and real-world studies [21,24]. Similar trends were also observed in patients treated with donanemab in clinical trials [7]. The Asian subpopulation of Clarify AD showed lower ARIA rates despite comparable numbers of *APOE* ε4 carriers to the overall population (Asian subpopulation: 72.6 % [13]; overall population: 68.9 % [6]). Chen et al. [13] discussed that regional factors, such as a potentially lower prevalence of cerebral amyloid angiopathy in Asia regions, may provide a possible explanation. An earlier study found that *APOE* ε4 carriers are less prevalent in Asia compared to Europe and North America [26]. Seemingly lower ARIA rates in our post-marketing study may, in part, reflect regional differences in cerebrovascular pathology and the prevalence of *APOE* ε4 carriers [6,13]. Additionally, the relationship between *APOE* status and ARIA incidence, particularly ARIA-H in real-world studies shows variability. For example, Paczynski et al. [21] reported that isolated ARIA-H was not associated with the number of *APOE* ɛ4 alleles. As we report interim findings, further follow-up data would clarify these points.

At the time our study was initiated, *APOE* biomarker testing was not covered by national insurance, and it appears that a small proportion of patients participated in this study underwent such testing. As *APOE* status may further increase ARIA risk in patients treated with monoclonal antibodies, biomarker findings would help guide clinical practice for physicians/healthcare professionals and assist patients/care persons in making informed decisions before initiating treatment [27].

The proportion of patients who experienced infusion-related reactions during follow-up was 17.0 %, and serious cases were observed in 18 patients (0.7 %). In an 18-month Clarity AD [6], infusion-related reactions occurred in 26.4 % of overall participants (237/898) and in 12.3 % of the Asian subpopulation (18/146) [13]. The observed number appears to be lower than that reported in clinical trials and real-world studies [21,22] but is comparable to the findings from the Asian subpopulation. Additionally, in our study, infusion-related reactions predominantly occurred within seven days of the first infusion and, across infusions, they typically occurred on the day of infusion. These findings are again similar to those reported in Clarity AD [6]. Shields et al. [22], using real-world data, described the distribution of occurrence across infusions, and 37 % occurred after the first infusion, 7 % after the 2nd, 4 %–6 % after the 3rd–7th, and none after the 8th–14th. These findings altogether suggest that particular caution is required after the first infusion.

This study has several limitations. It was conducted in patients treated with lecanemab in routine clinical settings in Japan, and the findings should be interpreted in the context of the study population and setting. In this observational study, patients are diagnosed with MCI and mild dementia due to AD, and the presence, absence, and severity of MRI abnormalities are assessed at the discretion of physicians at participating institutions without central review. MRI readings are performed by different radiologists and physicians at each institution (however, all radiologists and physicians are expected to complete the web-based training). Furthermore, unlike clinical trials that reported adverse events regardless of causality [6], this study reports ADRs, thereby precluding direct numerical comparison with clinical trial safety profiles. In addition, we report the interim analysis findings from the subset of registered patients for whom CRF data were locked by the data cutoff (predominantly from CRF1 with a small proportion from CRF2). As inclusion in this analysis depended on the timing of data entry at each site, the analyzed subset may be subject to selection bias. Therefore, the incidence of ADRs and discontinuation rates may be underestimated or overestimated in this interim subset. It is important to note, however, that regulatory requirements mandate reporting of serious ADRs irrespective of whether the CRF data are locked. Nevertheless, the observed trends may change as more data become available. Comprehensive reports including changes in cognitive function and ADL will be provided in future reports.

While the study limitations should be acknowledged, it also has important strengths. A key strength is its design as an all-case surveillance targeting patients treated with lecanemab in Japan, thereby minimizing selection bias. This study included patients registered at medical facilities across Japan and represents one of the largest real-world cohorts reported globally to date. By drawing upon real-world data rather than those from clinical trial settings, this interim analysis describes safety profiles over approximately six months in patients with diverse patient characteristics. In addition, preliminary findings from genotype-stratified analyses provide clinically relevant insights for patients with a genetic risk of ARIA.

## 5 Conclusion

Over the 189.6-day lecanemab treatment, the proportion of patients who experienced ARIA as an ADR was 7.1 %, and those of ARIA-E and ARIA-H were 3.0 % and 5.2 %, respectively, with the majority of cases being asymptomatic. The proportion of patients who experienced ARIA was highest in *APOE* ε4 homozygous patients, and although absolute rates are not directly comparable with those from clinical trials, the trends in ARIA distributions across *APOE* genotypes among patients confirmed to have *APOE* genotypes were nearly identical to those found in clinical trials. Infusion-related reactions were within the range observed in clinical trials. Comprehensive safety and effectiveness data from extended treatment will be provided in future reports.

## Funding

This post-marketing study was funded by Eisai Co., Ltd. and Biogen Inc. Eisai was involved in the study design, conduct of the study, analysis and interpretation of data, and preparation and approval of the manuscript. Medical writing support was provided by Clinical Study Support, Inc., which was also funded by Eisai Co., Ltd.

## Data statement

The data used in this study are not publicly available due to privacy and ethical reasons.

## Declaration of generative AI and AI-assisted technologies

No generative AI or AI-assisted technologies were used in the writing process or in creation of figures.

## CRediT authorship contribution statement

**Atsushi Iwata:** Writing – review & editing, Writing – original draft, Supervision, Methodology, Investigation, Conceptualization. **Yukinori Sakata:** Writing – review & editing, Writing – original draft, Visualization, Formal analysis. **Kinuyo Koizumi:** Writing – review & editing, Project administration, Methodology, Investigation, Formal analysis, Data curation, Conceptualization. **Akira Endo:** Writing – review & editing, Project administration, Methodology, Investigation, Formal analysis, Data curation, Conceptualization. **Weijie Kuang:** Writing – review & editing, Project administration, Methodology, Investigation, Formal analysis, Data curation. **Kenta Sumitomo:** Writing – review & editing, Project administration, Methodology, Investigation, Conceptualization. **Mika Ishii:** Writing – review & editing, Supervision, Resources, Funding acquisition.

## Declaration of competing interest

The authors declare the following financial interests/personal relationships which may be considered as potential competing interests:

Atsushi Iwata received consulting or advisory fees from Eisai Co., Ltd., Otsuka Pharmaceutical Co., Ltd., Fujirebio Inc., Janssen Pharmaceuticals Inc., Eli Lilly and Company, Biogen Inc., and Chugai Pharmaceutical Co., Ltd. The author received speaking and lecture fees from Otsuka Pharmaceutical Co., Ltd., Daiichi Sankyo Inc., Kowa Co., Ltd., Fujirebio Inc., Eli Lilly and Company, Biogen Inc., and Sysmex Corporation. The author received research fundings from Fujirebio Inc., Janssen Pharmaceuticals Inc., Eli Lilly and Company, Biogen Inc., Kobayashi Pharmaceutical Co., Ltd., Chugai Pharmaceutical Co., Ltd., Sony Group Corporation, Sysmex Corporation, and FUJIFILM Corporation. Yukinori Sakata, Kinuyo Koizumi, Akira Endo, Weijie Kuang, Kenta Sumitomo, and Mika Ishii are employees of Eisai Co., Ltd.

## Appendix Supplementary materials

Image, application 1
