# Supplementary material for: Interim analysis of all-case post-marketing surveillance study in Japan: lecanemab in patients with early Alzheimer’s disease
Source: J Prev Alzheimers Dis. 2026 Mar 17;13(5):100541. doi: 10.1016/j.tjpad.2026.100541 (PMC13014925; doi:10.1016/j.tjpad.2026.100541)
Supplement: Supplementary file 1 [file mmc1.docx]

# **Supplementary material**

## **eFigure 1** Patient disposition


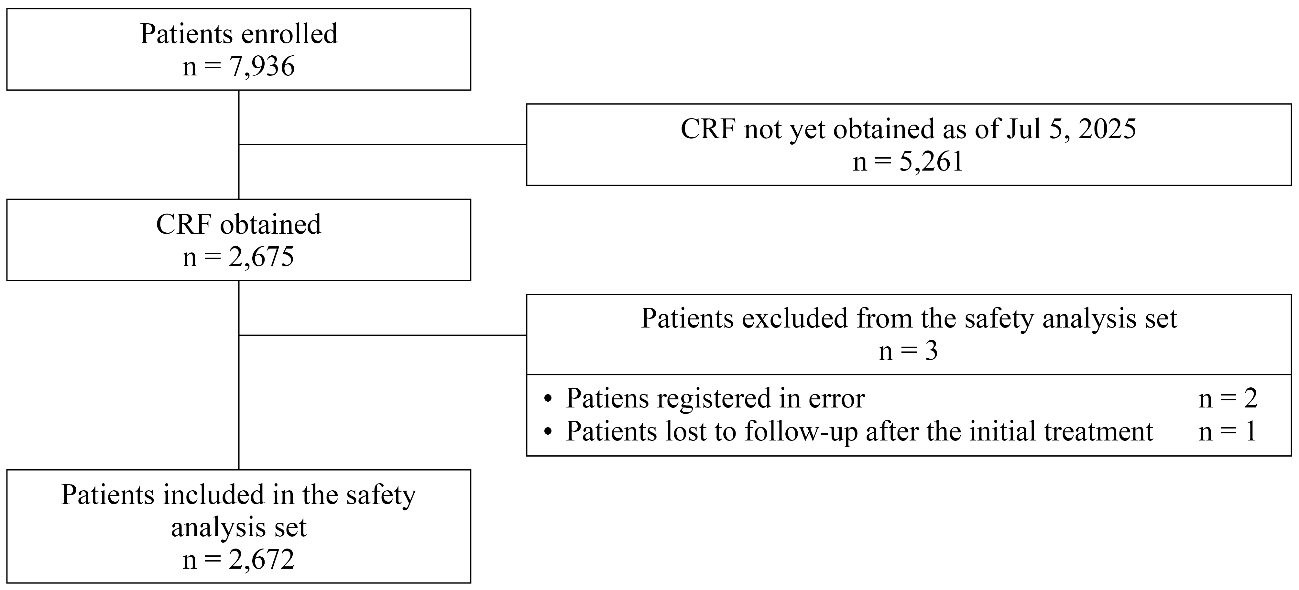


**Abbreviations:** CRF, case report form.

**Notes:**

Patients included in the present interim analysis report represent a portion of the enrolled patients whose CRFs have been obtained.

## **eFigure 2** Adverse drug reactions: ARIA by *APOE* ε4 status


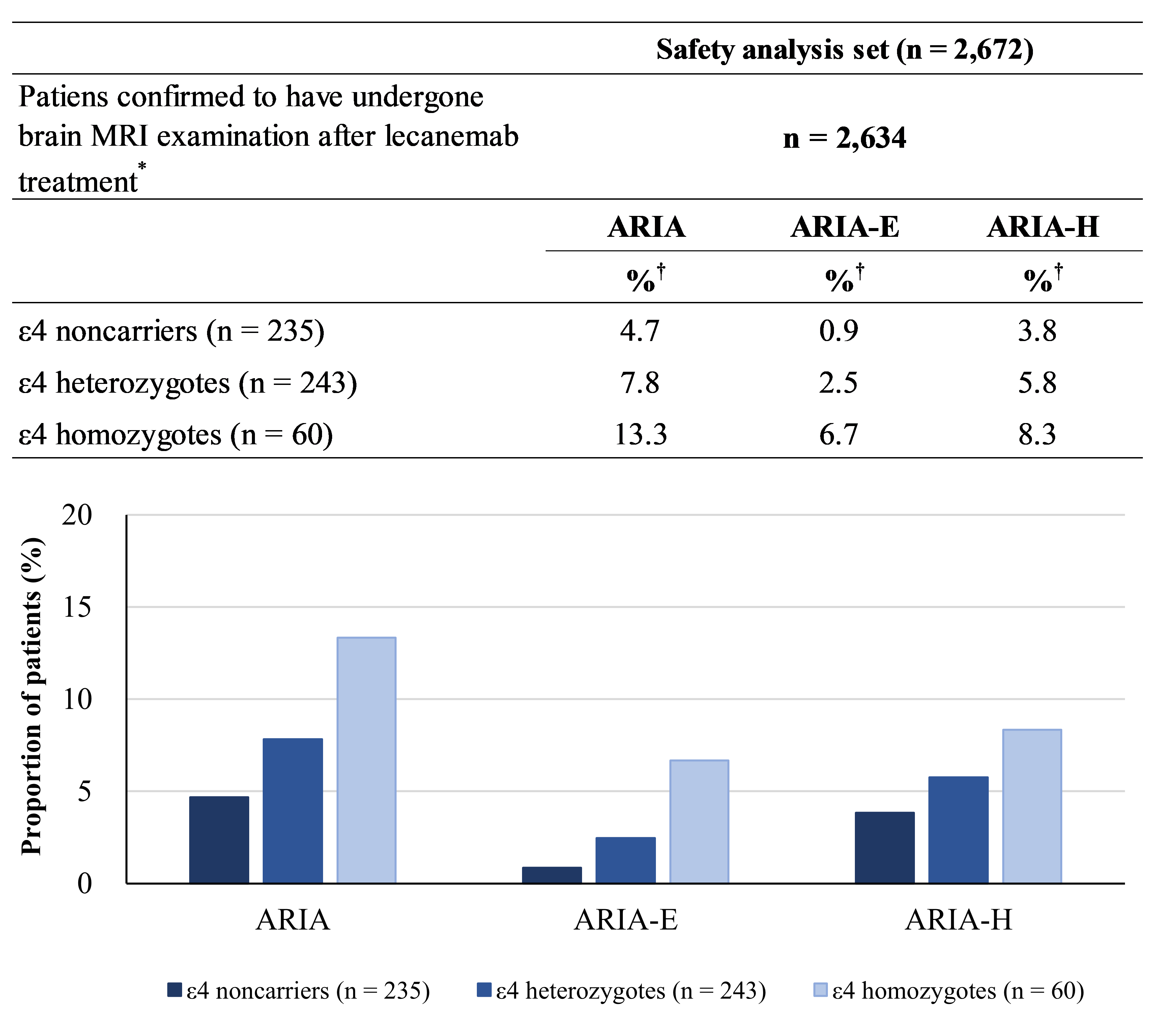


**Abbreviations:** ARIA, amyloid-related imaging abnormalities; *APOE*, apolipoprotein E; MRI, magnetic resonance imaging; ARIA-E, ARIA with edema or effusions; ARIA-H, ARIA with cerebral microhemorrhages, superficial siderosis, and macrohemorrhage.

**Notes:**

^*^ Even if it was not possible to confirm brain MRI examination performed for patients after the start of treatment, patients were included if they had developed ARIA.

^†^ The denominator is the patients described in brackets. Fourteen patients whose *APOE* test findings were not shared or not yet available and 2,082 patients who did not undergo *APOE* genotype testing are not included in the figure.

## **eFigure 3 Cumulative incidence of first adverse drug reactions: a) ARIA, b) ARIA-E, and c) ARIA-H, stratified by *APOE* ε4 status**


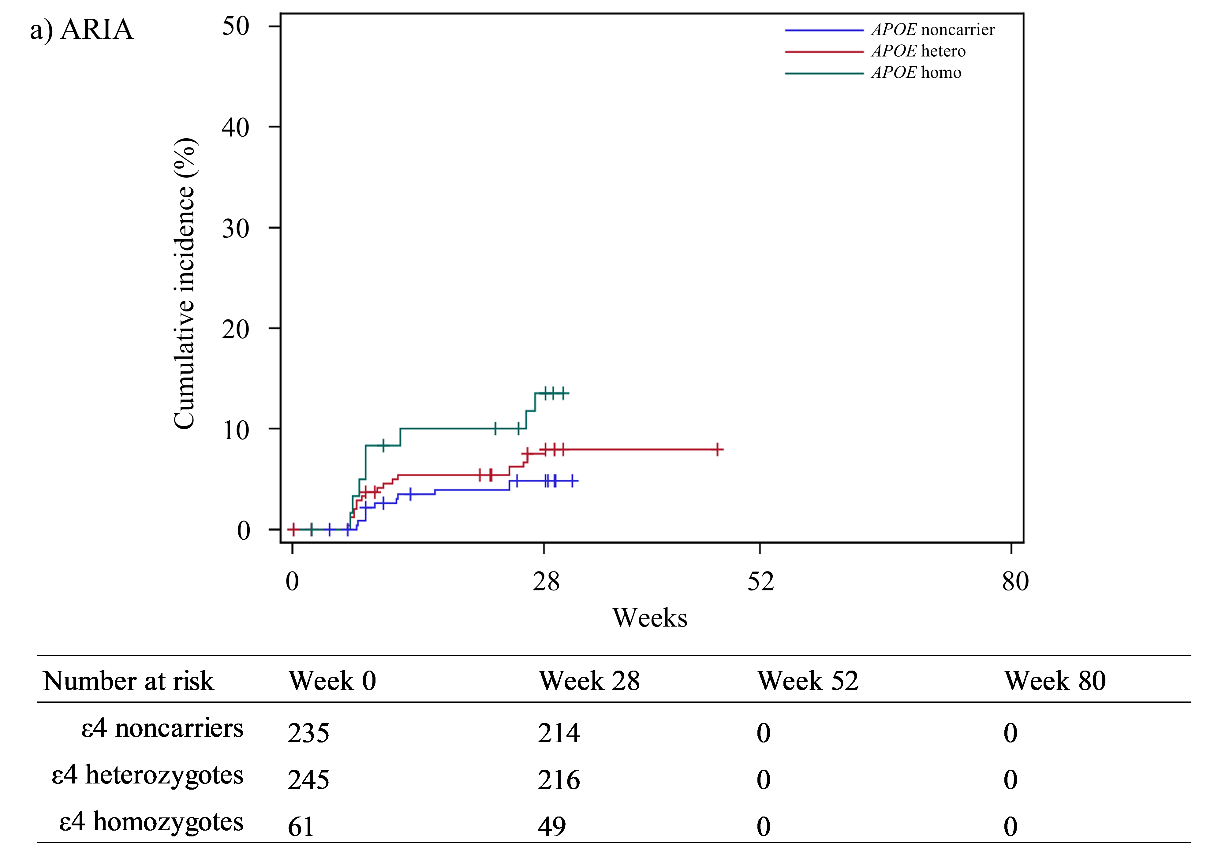


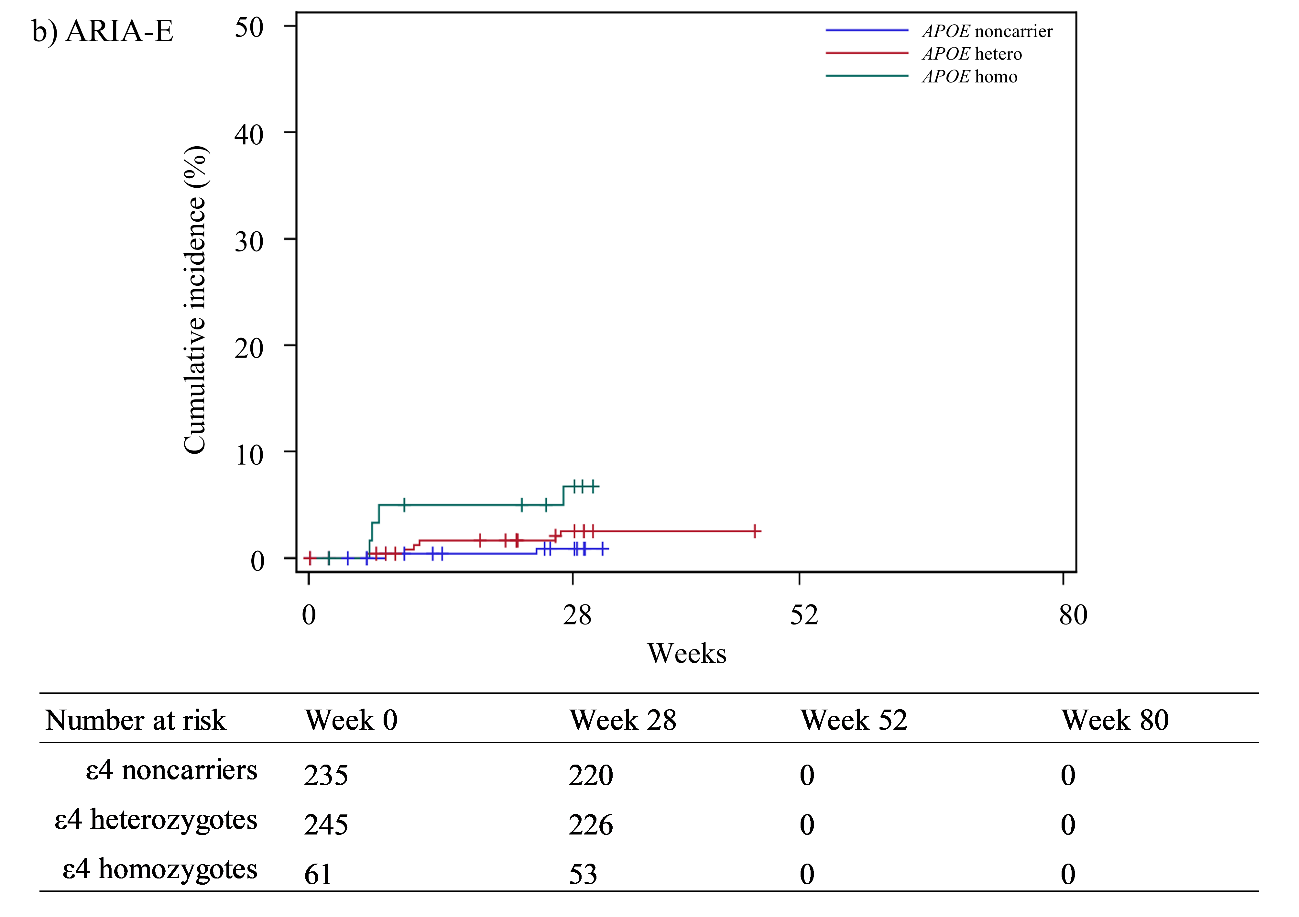


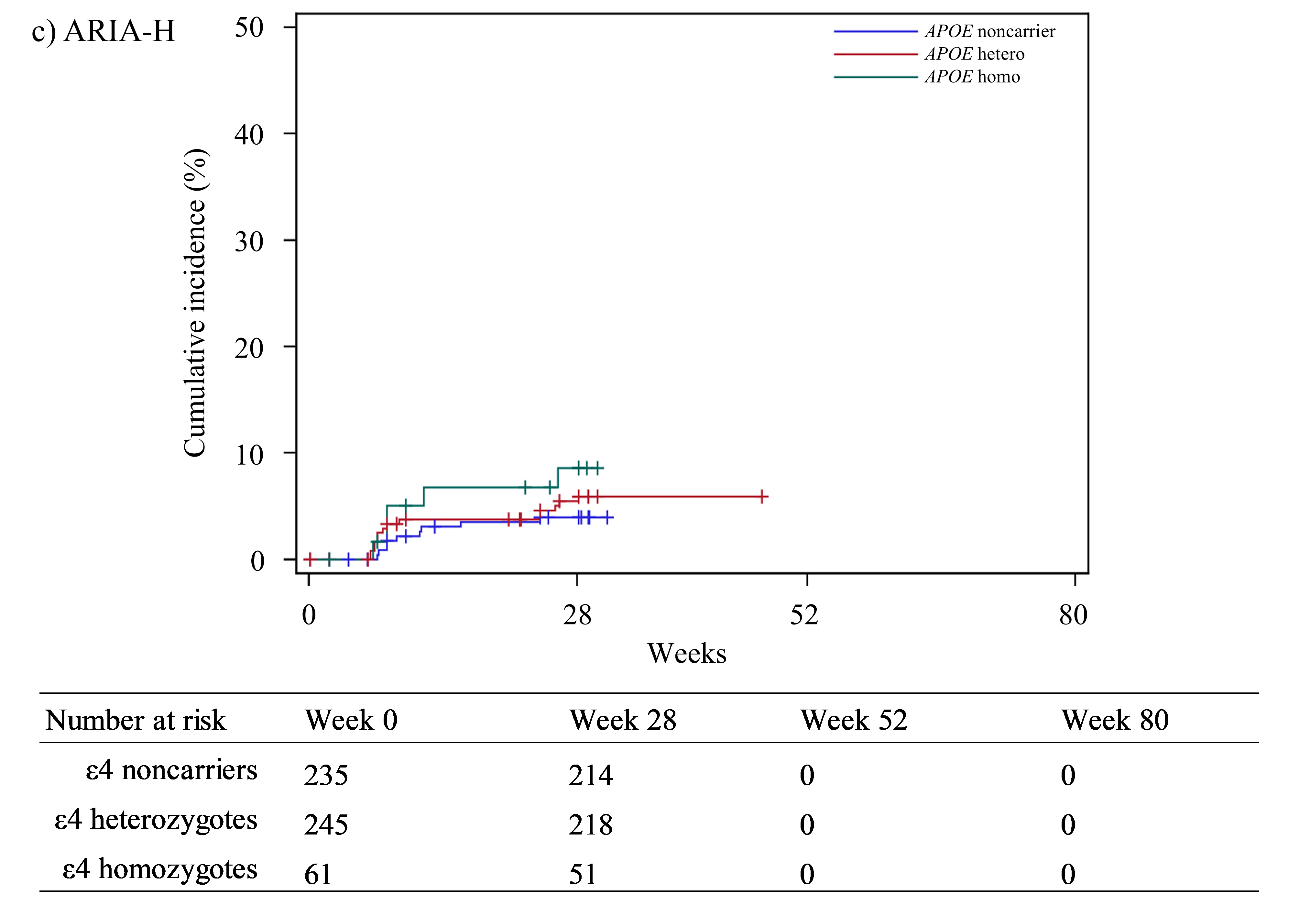


**Abbreviations:** ARIA, amyloid-related imaging abnormalities; ARIA-E, ARIA with edema or effusions; ARIA-H, ARIA with cerebral microhemorrhages, superficial siderosis, and macrohemorrhage; *APOE*, apolipoprotein E.
